# Transcription Factors with Targeting Potential in Gliomas

**DOI:** 10.3390/ijms23073720

**Published:** 2022-03-28

**Authors:** Angeliki-Ioanna Giannopoulou, Dimitrios S. Kanakoglou, Christina Piperi

**Affiliations:** Department of Biological Chemistry, Medical School, National and Kapodistrian University of Athens, 11527 Athens, Greece; angelig@med.uoa.gr (A.-I.G.); kanakogloud@biol.uoa.gr (D.S.K.)

**Keywords:** transcription factors, GLI, E2F, STAT3, HIF-1/2, NFI-A/B, TBXT, MYT1, TMZ, gliomas, therapy

## Abstract

Gliomas portray a large and heterogeneous group of CNS tumors, encompassing a wide range of low- to high-grade tumors, as defined by histological and molecular characteristics. The identification of signature mutations and other molecular abnormalities has largely impacted tumor classification, diagnosis, and therapy. Transcription factors (TFs) are master regulators of gene expression programs, which ultimately shape cell fate and homeostasis. A variety of TFs have been detected to be aberrantly expressed in brain tumors, being highly implicated in critical pathological aspects and progression of gliomas. Herein, we describe a selection of oncogenic (GLI-1/2/3, E2F1–8, STAT3, and HIF-1/2) and tumor suppressor (NFI-A/B, TBXT, MYT1, and MYT1L) TFs that are deregulated in gliomas and are subsequently associated with tumor development, progression, and migratory potential. We further discuss the current targeting options against these TFs, including chemical (Bortezomib) and natural (Plumbagin) compounds, small molecules, and inhibitors, and address their potential implications in glioma therapy.

## 1. Introduction

Gliomas represent the majority (80%) of all primary malignant central nervous system (CNS) neoplasms, affecting both the brain and spinal cord. Primary CNS tumors occur more frequently in adults (29 per 10,000) than in children and adolescents; however, they are the most common types of solid tumors among pediatric cancers. According to histological similarities and cell origins, gliomas are divided to glial (astrocytomas, oligodendrogliomas, and ependymomas) and non-glial (meningiomas and medulloblastomas) tumors. The distinct types of gliomas range from grade I to IV regarding their aggressiveness and proliferative potential, with grade IV corresponding to the most malignant phenotype [1,2,3].

The pathogenesis of gliomas has been linked to several genetic alterations and deregulation of major signaling pathways. These defects include homozygous deletion of the *cyclin-dependent kinase inhibitor 2A (p16)* gene and complete chromosome 1p/19q deletion. They also involve mutations in tumor-suppressive genes such as *Neurofibromatosis type 1* *(NF1)*, *Phosphatase and Tensin homolog (PTEN)*, *tumor protein p53 (p53)*, and *Retinoblastoma (RB)* and in genes that are associated with metabolism and telomere length maintenance, like *Isocitrate Dehydrogenase* (*IDH*) isozyme genes and *Telomerase Reverse Transcriptase* (*TERT*), *alpha-thalassemia/mental retardation, X-linked* *(ATRX),* and *Death domain Associated protein* (*DAXX),* respectively. The signaling axis RAS/RAF/MEK is commonly dysregulated in certain types of gliomas, with a mutation in serine/threonine protein kinase BRAF where valine is substituted with glutamic acid at amino acid 600, affecting cell growth and differentiation. In addition to genetic changes, epigenetic alterations involving DNA methylation, histone modifications, and miRNAs have emerged in the last few years as important contributors to neoplastic transformation and progression due to their interplay with gene expression [3,4,5,6]. In particular, mutations affecting *IDH* genes result in the production of the natural metabolite α-ketoglutarate and the oncogenic byproduct, 2-hydroxyglutarate (2HG) [7]. The accumulation of 2HG leads to global DNA hypermethylation by restricting the function of TET enzymes, which are known demethylases. This swift DNA methylation pattern interferes also with the binding and activity of several transcription factors (TFs). Depending on the factors’ protein domains and corresponding motifs, the activity and binding site recognition ability of some TFs are repressed, while, in others, these features are promoted by DNA methylation [8]. In this way, epigenetic events, such as DNA methylation, may jeopardize gene expression programs.

The World Health Organization Classification, 2021 edition (hereafter, WHO 2021) on gliomas has been updated in order to encompass information on tumors’ phenotypic and genotypic profiles and improve the diagnostic and prognostic accuracy. The standard therapeutic approach for gliomas combines surgery, radiation, and chemotherapy with alkylating agents. Although, in some cases, therapy is beneficial, the most malignant types like glioblastoma (grade IV) exhibit recurrence and significant mortality. These properties are associated with a combination of biological, genetic, and signaling alterations that confer to tumor heterogeneity and diverse patient responses to therapy. Of great significance are specific cell niches inside the tumor, known as glioma stem cells (GSCs), that confer to this heterogeneity. Consequently, there is a mandatory need for the development of targeted molecular therapies and personalized therapeutic approaches [3,9].

Intracellular signaling pathways share a converging point in the nucleus where activation of specific transcription factors takes place. Gene expression is governed by the interplay between *cis*-regulatory elements, such as promoters, enhancers, silencers, and trans-acting factors, including TFs. Transcription factors most commonly bind directly to specific sequences on their target gene promoters but can also affect promoter activity by localizing to distal enhancer regions. These interactions evoke an increase or decrease in gene expression, affecting the protein synthesis rate and, ultimately, tailoring cellular behavior. To date, several mechanisms that lead to the deregulation of TFs have been reported in a wide range of cancers. Both indirect means, such as aberrant activity or mutations in upstream signaling molecules and cofactors, and direct means, such as deletions, amplifications, rearrangements, gain or loss-of-function point mutations in genes encoding TFs, contribute to altered function and expression of these regulatory proteins in cancer. In the aftermath of TF deregulation, a series of events depicted as hallmarks of cancer arise, which subsume uncontrolled cell proliferation, immune evasion, establishment of a stem cell-like phenotype, epithelial to mesenchymal transition (EMT), the prevention of cell death pathways, and therapeutic resistance.

Drug repositioning and novel therapeutic agents are imperative for the future treatment of gliomas, as there is an urgent need for treatments that could improve the GBM prognosis. In this review, we address the role of specific transcription factors in glial tumors based on their involvement in the pathogenesis of gliomas, their utility as biomarkers, and pharmacological targeting potential [10].

Several oncogenic TFs that belong to the GLI, E2F, STAT, HIF, FOXM, and ATF families, as well as several tumor-suppressive TFs of the NFI, T-box, and NZF families, have been selected. Current applications and future perspectives of these TFs as targeting options in the management of gliomas are critically discussed.

## 2. Oncogenic Transcription Factors

Several TFs have been allocated an oncogenic role in gliomas either through deregulated expression or altered function due to fusion with other proteins, eventually affecting cell proliferation, differentiation, and apoptosis. In this section, we discuss experimental evidence on the oncogenic role of GLI, E2F, STAT, HIF, FOXM, and ATF family members and current targeting options.

### 2.1. GLI Transcription Factors

The Glioma-Associated Oncogene (GLI) transcription factor family consists of three members, GLI-1, -2, and -3, all of which contain conserved tandem C_2_H_2_ zinc finger domains and a consensus histidine/cysteine linker sequence between zinc fingers [9]. They recognize the GACCACCA consensus sequence on promoters of their target genes, including *CDC2*, *hTERT*, *IRIS1*, *FOXM1*, and *BMI1*, via the zinc finger motifs of their DNA-binding regions [11,12,13,14,15].

All members of the family are canonically activated by a multiprotein cascade involved in Hedgehog (Hh) signaling in order to regulate transcription of Hh target genes, such as *PTCH1*, *PTCH2*, and *GM1*. The Hh pathway plays a vital role in embryonic development, as it participates in the transmission of information to embryonic cells required for proper cell differentiation.

The regulation of the Hh signaling pathway relies on the balance between the activator and repressor forms of GLI transcription factors. Key components of the signaling cascade are the Hedgehog ligands (sonic Hh, Indian Hh, and desert Hh); Patched Receptors (PTCH1 and PTCH2); Smoothened Receptor (Smo); Suppressor of fused homolog (Sufu); protein kinase (PKA); and cyclic adenosine monophosphate (cAMP) [16]. All components of the signal transduction pathway have been detected in the primary cilia (PC) [17]. Upon absence of the Hh ligand, PTCH localizes at the PC base and suppresses the activity of Smo by inhibiting its translocation to the PC [18]. This results in the proteolytic cleavage of full-length glioma-associated oncogene (GliFL) and production of the Gli repressor (GliR) upon phosphorylation by PKA, glycogen synthase kinase-3 (GSK3), and casein kinase 1 (CK1) [19]. Subsequently, GliR binds to Hh target genes promoters, keeping them inactive. On the other hand, the binding of Hh to the PTCH1 receptor activates the signaling cascade. As a result, Smo inhibition is abrogated, and the signal gets transmitted via a cytoplasmic protein complex composed of Kif7, GliFL, and Sufu. Smo moves to the tip of PC and signals Sufu to release the Gli activator (GliA), which migrates into the nucleus and enhances gene transcription [16,20].

Deregulation of the Hh pathway, mostly activation, due to mutations at the associated genes or alterations in the expression of the signaling molecules, has been associated with developmental anomalies and various stages of carcinogenesis in different types of tumors. The key regulators of the pathway, GLIs, were first isolated from human glioblastoma cells in 1987. Since then, research advances have pointed that the expression of several Hh cascade components, such as GLI factors, PTCH, and Smo, were detected in several tumors of the nervous system, including gliomas. Their expression has also been correlated with poor prognosis of patient survival [21,22].

Among the three members of the GLI family, GLI1 is the best studied and associated to epigenetic modifications, since it has been shown to recruit histone acetyltransferase PCAF, inducing an active chromatin state on Hh target genes by increasing the H3K9 acetylation levels. GLI1, along with its truncated homolog (TGLI1), which behaves as gain-of-function GLI1, were reportedly shown to mediate angiogenesis in gliomas by targeting the *VEGF, MMP2, MMP9*, *VEGF-C, TEM7*, and proangiogenic *heparanase* (*HPSE*) genes, respectively [23,24,25,26]. The second member of the family, GLI2, was found to induce *CDK6* expression by binding to its promoter, thereby mediating cell proliferation in Hh-associated medulloblastoma genetic mouse models [27]. In another study, GLI1-3 expression, along with its target genes, *FOXM1* and *BMI1*, were present in all the tested glioma cell lines in contrast to normal brain tissue that lacked GLI1 expression. Moreover, GLI2 expression has been strongly linked to many types of glial tumors, including astrocytomas, gangliogliomas, glioblastomas, ependymomas, and oligodendrogliomas, whereas GLI1 and 3 correlated preferably with oligodendrogliomas. In addition, the *GLI1* expression levels were particularly high in grade III and IV gliomas, whereas *GLI2* was found overexpressed only in grade III tumors. At the same time, *GLI1-2* overexpression in these tumors was suggested to impact their progression, since high-grade gliomas patients exhibited worse survival rates [28]. Finally, it is evident that GLI factors play an important role in stem cell phenotype formation by sustaining the expression of related genes, such as *OCT4* or *SOX2* [29].

The targeting of GLI proteins is difficult, because their binding domains constitute a limiting parameter for the design of small repressive molecules against them [30]. Nevertheless, GLI antagonists GANT-61 and -58 and Arsenic Trioxide (As_2_O_3_) have been developed but, to our knowledge, have not been tested in gliomas yet [31,32,33]. Some compounds targeting the Hh pathway show promise in the treatment of Medulloblastoma (MB) by overcoming the frequent phenomenon of mutation-driven drug resistance that SMO antagonists face. These compounds are effective towards both the Hedgehog pathway and the bromodomain-containing protein 4 (BRD4). This function leads to an indirect restriction of GLI activity, since BRD4 has been reported to interact with *GLI1* and *GLI2* promoter regions through its bromodomains and affect, in a certain amount, their expression. Liu et al. optimized the structure of 4-Aryl-1,6-dihydro-7H-pyrrolo[2,3-c]pyridin-7-one 2 (ABBV-075), among other BRD4 nonspecific inhibitors that also exhibited Hh pathway restrictive potential. Consequently, they generated a derivative compound 25 by fusing a fluoro substituent at the C3 position of the pyrrole core and compound 35, with 4-methylcyclohexyl amino ousting the phenylether motif. Both molecules were shown to be efficient GLI inhibitors, while compound 25 was further shown to abrogate tumor growth in vivo [30].

### 2.2. E2F Transcription Factors

The cyclin-dependent kinase (CDK)-Rb-E2F axis directs cell cycle progression, overseeing the timing and integrity of genetic material replication. Critical regulators of the pathway are members of the E2F transcription factor family. This family can be divided into three groups according to the structure and function of its members: activators (E2F1–3A), canonical repressors (E2F3B–6), and atypical repressors (E2F7 and E2F8) [34]. The levels of activator proteins peak during the G1-S phase transition, whereas atypical repressor levels peak in the succeeding S phase. Canonical repressors are constitutively expressed during all the phases of the cell cycle [35].

**Figure 1:**
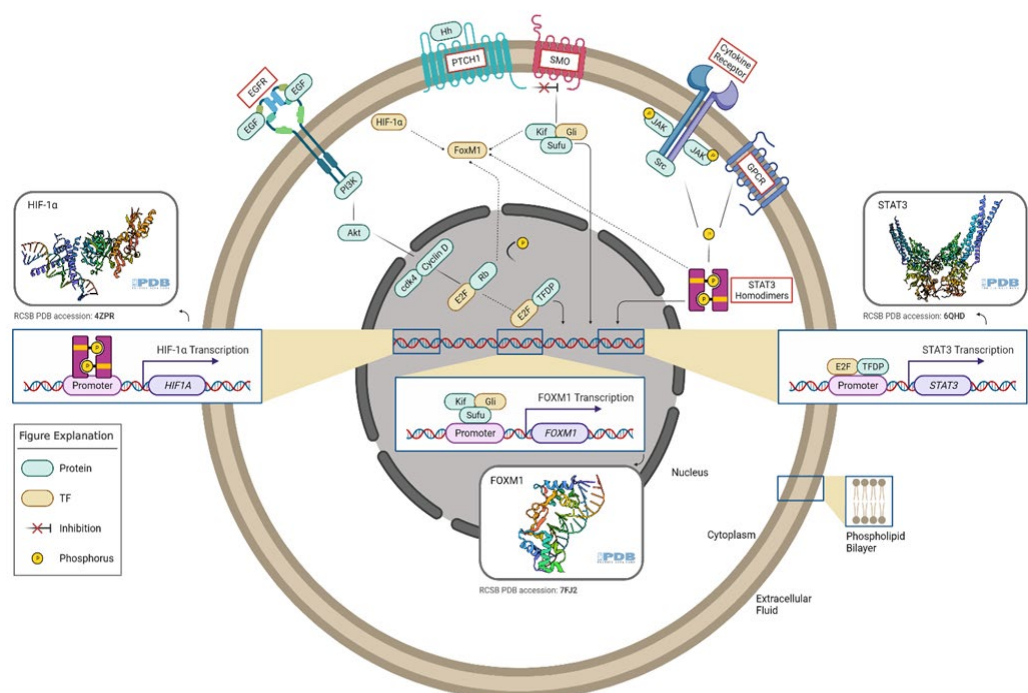
Oncogenic transcription factors and associated signaling pathways in gliomas. E2F TF is a downstream factor of the EGFR/PI3K/Akt pathway. The Rb tumor suppressor protein (pRb) binds to the E2F1 transcription factor, preventing it from interacting with the cell’s transcriptional machinery. When pRb gets phosphorylated, it detaches from E2F. E2F (along with its binding partner, TFDP) mediates the transactivation of E2F1 target genes, such as *STAT3*. GLI TF is a downstream effector of the Hedgehog pathway. In the absence of Hh, PTCH acts to prevent high expression and activity of SMO. GLI TFs function in a complex with Kif7 and Sufu, translocate to the nucleus, and induce the expression of target genes such as FoxM1. STAT3 TFs can be activated by several signals, which involve G-protein-coupled (GPCR) and cytokine receptors. Phosphorylated STAT3 homodimers regulate HIF-1α expression. FoxM1 can be activated by STAT3, GLI, HIF-1α, and E2F TFs. This figure was created with the tools provided by BioRender.com, accessed on 22 February 2022.

E2F factors contain a highly resembling winged helix DNA-binding domain (DBD) and share the ability to recognize and bind to the classic E2F consensus sequence TTCCCGCC (or slight variations of it) of their target gene promoters [36]. The DNA-binding ability of E2F1–6 transcription factors also depends on a dimerization (DIM) domain, which is composed of a leucine zipper (LZ) and a marked box (MB) domain [37]. To activate transcription, canonical E2Fs need to form a complex with a member of the transcription factor dimerization partner family (TFDP1, TFDP2 and TFDP3). E2F1–5 factors also carry a transactivation domain that binds pocket proteins (RB, p107, and p130) [38,39]. Upon RB presence, E2F activators are unable to promote cell cycle progression. On the contrary, E2F7 and E2F8, containing two tandem E2F DBDs, interact to form a single DNA-binding surface that recognizes the E2F consensus sequence independently of TFDP proteins (Figure 1) [40].

E2F factors and associated genes exhibit altered expressions in gliomas, according to a variety of studies. All E2Fs (except for E2F3 and E2F5) are highly expressed in high-grade gliomas (HGG) and linked to grade progression, indicating an adverse outcome [41]. Nonetheless, Li et al. portrayed a mechanism for glioma progression in their study, which involved the upregulation of *E2F3*. Overall, they demonstrated through several functional assays, MS2-RIP, and siRNA transfections that the lncRNA SNHG5 acts as an oncogenic factor in gliomas by competitively engaging (sponging) miR-205 and suppressing its function. Therefore, miR-205 is unable to bind its target sequences on *E2F3* 3′UTR, resulting in the upregulation of *E2F3* expression. They also showed that this mechanism drives glioma cell migration and invasion and increases glucose uptake in vitro, while its inhibition curtails tumor growth in vivo [42].

Regarding related genes, the upregulation of genes encoding DP family members has been reported alongside a significant rise in *E2F1* mRNA levels [43]. Moreover, Zhi et al. unveiled a potential mechanism by which ECT2 facilitates glioma cell proliferation both in vitro and in vivo. In their study, the ECT2 expression levels were increased in glioma cell lines and tissues compared to normal brain tissue and human astrocytes (NHAs) and correlated with the tumor grade. In summary, they suggested a pathway where ECT2 regulates the expression of PMSD14 deubiquitinase, which, in turn, stabilizes the E2F1 factors and prevents its degradation by proteasome machinery, resulting in *PTTG1* upregulation. Keeping in mind that PTTG1 can mediate glioma cell proliferation, the signaling cascade ECT2/PMSD14/E2F1/PPT11G could be potentially targeted as a therapeutic approach [44,45].

In the recent study of Yu et al., *E2F8* expression was found augmented in gliomas compared to normal brain tissues, especially in all four glioblastoma (GBM) subtypes (classical, mesenchymal, neural, and pro-neural), and associated with poor outcome regarding patients’ survival. Further investigation of E2F8 role in GBM revealed an attenuated proliferation of GBM cells and prolonged survival of animal models upon *E2F8* gene silencing. In addition, bioinformatic analysis pointed out a tight association of *E2F8* expression with aggressive cell cycle induction; DNA repair process; and key signaling pathways (STAT3, TGFRβ, and WNT). Moreover, the results from a correlation expression analysis and latter ChIP-PCR suggested E2F8 as a key candidate for *CHEK1* transcriptional activity regulation in GBM tumor cells. Collectively, these data demonstrate that E2F8 plays a pivotal role in cell proliferation, tumor formation, and multiple oncogenic processes in GBM [46].

Yang et al. investigated E2F7 role and function in gliomas and observed an upregulation of this TF in GBM patients, which was associated with poor overall survival. In vitro functional studies and in vivo model experiments revealed that E2F7 induced cell proliferation, cell cycle progression, and metastasis featuring tumorigenic abilities. Moreover, functional studies on E2F7 promotion of transcription and its participation in epigenetic mechanisms revealed that E2F7 binds to *EZH2* promoter, activating its transcription and increasing the H3K27me3 levels. Subsequently, EZH2 recruited H3K27me3 to *PTEN’s* promoter, inhibiting its expression and turning on the AKT/mTOR signaling pathway. Seemingly, E2F7 tumorigenic properties rely on the EZH2-mediated PTEN/AKT/mTOR pathway in GBM [40,47]. In addition, Lu et al. uncovered the role of lncRNA SNHG12, which has been found overexpressed in GBM cell lines and tissues as a mediator of cell proliferation and resistance to treatment with temozolomide (TMZ) in GBM. The overexpression of SNHG12 is attributed to a decline in DNA methylation at its promoter, which enables the engagement of SP1 transcription factor and, ultimately, transcriptional induction. Furthermore, the study demonstrated that miR-129-5p gets sponged by SNHG12, and its downregulation was involved in the promotion of TMZ resistance. As an outcome, *MAPK1* and *E2F7*, which carry binding sites for miR-129-5p at their 3′UTRs, were detected upregulated in TMZ-resistant GBM cells. Although the knockdown of both genes altered the resistant phenotype and cell proliferation rate, the E2F7 factor was mainly linked to G1/S transition, while MAPK1 is implicated in both G1/S transition and cell apoptosis with regards to TMZ treatment [48].

### 2.3. STAT3 Transcription Factor

Signal Transducer and Activator of Transcription 3 (STAT3) belongs in the family of STAT proteins composed of signal transducers and transcription regulators. The family encompasses seven members (STAT1, 2, 3, 4, 5A, 5B, and 6) that are encoded by different genes and exhibiting different functions but sharing a common structure [49]. The protein structure consists of six functional domains: an N-terminal, a coiled-coil (CC), a DBD, a linker sequence, Src Homology 2 (SH2), and finally, a transactivation domain (TAD). Of great significance are a tyrosine residue at amino acid position 705 (Tyr705) located in the SH2 domain and a serine phosphorylation site at residue 727 (Ser727) within the C-terminal domain, both involved in STAT activation [50].

The gene encoding *STAT3*, the third member of the family, resides at the 17q21.31 genomic region. To date, two isoforms of STAT3, the full-length STAT3α (770aa) and the truncated STAT3β (722aa), have been identified. They arise from alternative splicing and proteolytic cleavage processes. Interestingly, the truncated forms of STAT proteins, such as STAT3β, act mainly as dominant-negative of the corresponding full-length proteins [51,52].

STAT3 activation can be induced by extrinsic and intrinsic stimuli associated with cytokine signaling; some plasma membrane receptors (EGFR and PDGFR); and cytoplasmic kinases (Src family, BMX, and Bcr-Abl fusion protein), respectively (Figure 1). The phosphorylation of Y705 residue at the carboxyterminal is the most frequent of STAT3 modifications and is considered as its canonical activation marker. This can be achieved either by recruitment of JAK kinases to the receptor’s cytoplasmic tail or directly by specific cytoplasmic kinases. Additional posttranslational modifications of STAT3 that lead to its activation include phosphorylation at Ser727, acetylation (at lysine residues K49 and K87), and methylation (at lysine residue K140) [53,54,55]. STAT3 can be regulated by several mechanisms, including a synthesis/degradation cycle dependent on proteasome function and an activation/inactivation loop, mostly linked to its phosphorylated/dephosphorylated state (Figure 1).

Upon phosphorylation, STAT3 undergoes dimerization via reciprocal interactions with SH2 domains that bind to phosphotyrosine. Thereafter, the formed active homo- and heterodimers can translocate to the nucleus and stimulate transcription through recognition of the small palindromic consensus sequence TTCN_2-4_GAA that defines GAS elements within target gene promoters [48].

Of all members of the family, STAT3 is most frequently implicated in various types of cancers [53]. The deregulation and constant activation of STAT3 in gliomas is considered to result from an aberrant signal from upstream regulators, since no gain-of-function mutation of this molecule has been identified yet. On the one hand, this speculation concerns gain-of-function mutations or enhanced activation of an upstream activator, while on the other hand, it entails loss-of-function mutations or reduced activation of an upstream repressor [49]. These further lead to alterations in signaling pathways mediated by receptor-associated tyrosine kinase activities where growth factor receptors and cytokines are key components and upregulation of protein serine/threonine kinases.

Abnormally redundant signaling that occurs from gene amplifications and/or rearrangements of *EGFR* gives rise to the formation of a truncated variant (*EGFRvIII*) or of the fusion mutant *EGFR-SEPT14*, resulting in a hyperactive pSTAT3-Y705 molecule. Puram et al. demonstrated that STAT3 promotes transcriptional regulation of inducible nitric oxide synthase *(iNOS)* in GBs, which specifically carry the activated *EGFRIII* variant. STAT3 was associated with tumor progression and invasive aptitude [56,57]. Another study focusing on TGF-β, a multifunctional polypeptide growth factor, showed that TGF-β-related glioma cells invasion required phosphorylation of STAT3 at the Y705 residue via IFITM3-STAT3 axis [58]. Moreover, cytokines IL-6 and OSM have been detected overexpressed in gliomas. Both cytokines induce STAT3 phosphorylation at Tyr Y705 through the hexameric receptor complex IL-6Rα. In particular, OSM contributes to the aggressiveness of the mesenchymal subtype and has the ability to activate STAT3 expression by forming a complex with EGFRvIII, which, at the end results, in its overexpression.

It is also notable that activation of STAT3 in GBM stem-like cells has been linked to the activity of non-tyrosine kinases, like the bone marrow and X-linked (BMX) nonreceptor tyrosine kinase [49,59,60]. Serine/threonine kinases mediate STAT3 phosphorylation at serine 727 residue. For instance, PKCε (Protein Kinase C epsilon) overexpression in human anaplastic astrocytoma and GBM cases seems to consort constitutive activation of STAT3 through serine 727 phosphorylation [61,62].

At the same time, deregulation of *STAT3* expression may also refer to the deficiency of upstream repression regulators, such as PIAS3, SOCS, and PTPRD. For instance, a reduced expression of PIAS3 accompanied by elevated pSTAT3-Y705 levels has been observed in GBM, in contrast with normal brain tissues [48,63].

Regardless of the mechanism behind its activation, STAT3 undoubtedly possesses a crucial part in the pathogenesis of gliomas, the proliferation and migration of glioma cells, while contributing to the stem-like phenotype, angiogenesis, and immune suppression. Regarding cell survival and proliferation, several in vivo and in vitro approaches that focus on STAT3 inhibition have demonstrated a mitigated accumulation of antiapoptotic factors, such as Survivin, Bcl-2, Bcl-Xl, and Mcl-1, and a concomitant attenuated expression of cell cycle regulators, like c-myc, cyclin E, and cyclin D1 [64,65]. Besides cell proliferation, STAT3 has been linked to migration and invasion of glioma cells. According to some studies, inhibition of STAT3 led to reduced production of matrix metalloproteinases (MMP2 and MMP9) and was associated with genes that account for EMT, namely *Snail* [66]. In addition, STAT function is associated with p65-NF-*κ*B and nuclear factor I-X3, resulting in the upregulation of *ICAM-1* and *YK1-40*, respectively, fueling the migration and invasion abilities of glioma cells [67].

Moreover, STAT3 activation due to plasma membrane and cytokine stimuli has been shown to induce immune tolerance. Its activation attenuates differentiation, maturation, and functions of dendritic cells; disrupts T-cell proliferation; and promotes T-cell anergy and immunosuppressive microglia [4,68,69].

Moreover, STAT3 transcription factor inhibition is considered as a promising approach for glioma treatment, mostly in GBM cases since it drives pro-neural–mesenchymal transition and is implicated in the aggressiveness and stemness of glioma tumors. The study of Tan et al. distinguished two subgroups based on a transcriptomic signature associated with the STAT3 pathway that could help to predict the patients’ response to therapy with STAT3 inhibitors. STAT3^high^ defined a patient cohort enriched in the mesenchymal and classical molecular subtypes with non 1p/19q codeletion and IDH-WT status, describing highly aggressive and recurrent gliomas. On the contrary, STAT3^low^ tumors are comprised mostly of low-grade gliomas (LGGs) and the pro-neural molecular subtype with enrichment of 1p/19q codeletion and IDH-mutant status, presenting tumors of better prognosis and responsiveness to current chemotherapy. They demonstrated that dual inhibition of IGF-1R with NT157 and STAT3 with AZD1480 and/or Linsitinib sensitizes STAT3-low cells and improves survival. The latter also functions synergistically with the TMZ standard treatment [70]. Likewise, JSI-124 (cucurbitacin I), a natural chemical compound, has been shown to suppress the expression of VEGF and blocked the phosphorylation of JAK2 in a dose-dependent manner. Thus, the antiangiogenic effects of JSI-124 might occur through VEGFR2/STAT3 (Ser727) inhibition [71]. Additionally, JSI-124 was shown to sensitize glioma cells to DNA-alkylating agents TMZ and cisplatin [72]. Other STAT3 pharmacological inhibitors, such as AG490, WP1066, LLL3, and Gefitinib, have also exhibited potential therapeutic benefits [73,74]. Especially WP1066, which explicitly targets glioma cells leaving intact normal astrocytes, can shift immune tolerance in glioma patients by inducing the production of costimulatory factors in macrophages that infiltrate glioma tumors and cytokines that trigger effector T cells [75]. Additionally, Ibrutinib (PCI-32765), which is an approved small molecule for the treatment of mantle cell lymphoma and chronic lymphocytic leukemia, has been shown to target BMX in GSCs and alleviate tumor expansion in GSC-derived orthotopic xenografts. Therefore, Ibrutinib constitutes an attractive option for the indirect inhibition of STAT3 hyperactivation in GBM [76]. Kadiyala et al. designed albumin-based nanoparticles (NPs) bearing the tumor-penetrating peptide iRGD to successfully infiltrate the blood–brain barrier, deliver specific small-interfering RNA (siRNA), and silence *STAT3* expression in GBM tumors. This is a very promising approach, since the NPs induced prolonged survival in synergy with ionizing radiation (IR) treatment and immunological memory against GBM recurrence in mice [77].

### 2.4. HIF Transcription Factors

The maintenance of oxygen homeostasis is crucial especially for organisms like metazoans, which rely mostly on aerobic energy production. Hypoxia-inducible transcription factors (HIFs) are key regulators of gene expression in hypoxic conditions featuring reduced oxygen levels. Genes that are activated upon oxygen reduction are those implicated in mitochondrial function, energy metabolism, oxygen binding, and delivery, as well as hematopoiesis [78,79]. HIFs are also responsible for the regulation of *VEFG* expression and may be involved in the formation of the endothelium that gives rise to the blood–brain barrier.

Structurally, HIFs is composed of two subunits that can form a functional heterodimer in order to regulate transcription. Three paralogs of the HIF-α (HIF-1α, HIF-2α/EPAS, and HIF-3α) and two paralogs of the HIFβ (ARNT and ARNT2) subunit have been detected [79]. The α-subunits are oxygen-responsive cytoplasmic proteins, whereas β-subunits are nuclear proteins expressed in a constant rate. The proteins of this family are defined by the existence of an N-terminal bHLH (basic helix-hoop-helix) DNA-binding domain upstream of two per-ARNT-Sim (PAS) domains [80]. The α-subunits may also contain an oxygen-dependent degradation domain (ODDD) serving as an inhibitory element and an N-terminal translocation domain (NTAD). In addition to the previous domains, HIF-1α and HIF-2α contain a C-terminal transactivation domain (CTAD) [81].

Under normal oxygen concentration, HIF α-subunits undergo degradation through hydroxylation by prolyl hydroxylase domain protein (PHD) and polyubiquination by Von Hippel-Lindau (VHL), assisted by the E3 ligase. The modified α-subunit is then degraded at the proteasome. On the contrary, during hypoxia, the activity of PHD is reduced resulting in the cytoplasmic HIF-α stabilization, accumulation, and translocation to the nucleus. There, the α-subunits dimerize with either one of the β-subunits assisted by bHLH and PAS domains. For HIFs to regulate gene transcription, bHLH domains must come into contact with the core nucleotides of HIF-responsive elements (HRE) within gene promoter regions and mediate their binding (Figure 1) [82].

Hypoxic conditions are considered as a common outcome of tumor progression and development among different cancer types, because cancer cells proliferate rapidly outgrowing the tumor’s blood supply. GBM tumors, probably because of their aggressive nature, are very keen to develop perivascular hypoxia. This is supported by immunohistochemistry that identifies HIF-2α expression in GBMs [83].

HIF-2α protein is closely linked to the stem phenotype of glioma cells, which is essential for tumor recurrence and resistance to therapy. The transcription factor is selectively upregulated in GSCs but absent in normal progenitor cells [84,85]. Although the mechanisms that underlie its upregulation are not completely understood, a recently identified gain-of-function missense mutation in the oxygen-dependent degradation domain may be a possible explanation, since it prevents its degradation [83]. HIF-2α expression has been linked to transmembrane CD44 glycoprotein produced by stem cells in the perivascular niche of GBMs. Functional studies, employing knockdown of the factor in GSCs, demonstrated decreased tumor sphere formation, reduced GSC-mediated angiogenesis (in vitro), induction of cell apoptosis, and repression of GSC oncogenes transcription. According to evidence from in vivo experiments, the knockdown of *HIF-2α* in glioma xenograft models increased survival and stalled the appearance of neurological impairment. Concomitantly, upon CD44 intracellular domain inhibition, a downregulation of *HIF-2α* and a containment of hypoxia-induced glioma stemness were observed [86,87,88]. Moreover, regarding patients’ survival, clinical trials, and REMBRANT database, an inverse correlation with HIF-2α expression was supported. In addition, an overexpression of HIF-2α was witnessed in several chemo-resistant cell lines [85,89].

HIFs also seem to drive the metabolic reprogramming of branched-chain amino acids (BCAAs) in GBM in response to hypoxia. BCAAs, including leucine, isoleucine, and valine, are transported to the cytosol by members of the L-type amino acid transporters family (LAT1-4) and catabolized by branched-chain aminotransferases BCAT1 and BCAT2. In GBM cells, HIF-1 and HIF-2 induce *LAT1* upregulation. In particular, HIF-1α solely mediates *BCAT1* transcription in GBM cells, notwithstanding that both proteins are able to bind directly to the HRE at the first intron of the *BCAT1* human gene. Additional evidence of HIF-mediated reprogramming of BCAA metabolism relies on the fact that knockout of *HIF1A* and *HIF2A* significantly reduced glutamate labeling of BCAAs in GBM cells in hypoxic conditions. Altogether, HIF family is important for cell homeostasis and its members have been risen as possible mediators of tumor progression [90].

Regarding the effects of hypoxic conditions in transcription of certain genes that promote the malignant properties of gliomas and neoangiogenesis, inhibition of HIF TFs and their signaling pathway components has caught the attention of the research community as a possible molecular therapeutic target. Specifically, Acriflavine (ACF), an FDA-approved small molecule, can be administered locally in the brain by penetrating the blood–brain barrier via biodegradable polymers and drive the apoptosis of glioma cells. The pathway leading to apoptosis involves the reduction of *HIF-1α* and its target genes (*PGK-1* and *VEGF*) expression, suggesting that HIF pathway inhibition drives ACF-mediated glioma cell death. These findings are of immense importance, since ACF results in almost 100% long-term survival, as confirmed by MRI and histological analysis [91]. In a comparable way, cyclic peptide inhibitor cyclo-CLLFVY and PT2385 or PT2977 interfere with the HIF-α/HIF-β dimerization process by interacting with the PAS domains of HIF-1α and HIF-2α, respectively [92]. Additionally, the topoisomerase inhibitor Topotecan attenuates tumor growth and angiogenesis through the inhibition of HIF-1α and its target genes expression in GBM in vivo models [93]. In accordance, a combinational treatment with Topotecan and Bevacizumab, a humanized monoclonal antibody against VEGF, has been reported to exert antiproliferative function towards glioma cells due to HIF-1α activity reduction [94]. Another novel small molecule, 103D5R, decreases *HIF-1α* expression, inhibits the transcription of HIF-1α target genes and prevents angiogenesis and metabolic adaptation in gliomas [95]. Moreover, the natural polyphenolic compound Vitexin was shown to repress *HIF-1α* expression. This flavonoid has been shown to comply with Hyperbaric oxygen (HBO) in increasing the sensitivity of glioma tumors to radiotherapy in mice [96]. In addition, Borneolum Syntheticum, commonly known as Borneol, is a bicyclic monoterpenoid reported to mediate apoptotic processes in glioma cells in vitro by overseeing *HIF1α* expression [97].

### 2.5. FOXM1 Transcription Factor

Forkhead Box M1 (FOXM1), also known as Trident, is a proliferation-specific factor that resides in the Forkhead box superfamily of proteins that share a preserved DNA-binding region. FOXM1 protein consists of three crucial for its function domains: the conserved winged helix DNA binding domain 1 (DBD), an N-terminal repressor domain (NRD), and a C-terminal transactivation domain (TAD) [98,99]. Regarding its arrangement in humans, the *FOXM1* gene contains ten exons. To date, four isoforms of FOXM1 have been established, which rise from alternative splicing between the Vα and VIIα exons. Despite their functional differences, all isoforms recognize and bind to the consensus sequence 5′-A-C/T-AAA-C/T-AA-3′ of their target genes, but only the latter three are transcriptionally active (Figure 1) [100,101,102].

*FOXM1,* in turn, is supervised at the transcriptional level by other transcription factors, which interact with *cis*-elements, E-boxes, and other regulatory elements contained, mainly, in its core promoter region. Transcription factors, including GLI1, CTCF, CREB, STAT3, E2F, and HIF-1a, act as activators, whereas LXRa and p53 function as repressors by direct binding to activating or repressing *cis*-elements of *FOXM1*, respectively. Additional elements, like Estrogen-Responsive Element (ERE) and E-box, can potentially bind both activating and repressive TFs, exhibiting a dual role in the regulation of *FOXM1* transcription. It is also worth noting that the FOXM1 protein can bind to the *FOXM1* promoter region during an autoregulatory loop. In regard to the posttranscriptional regulation of *FOXM1* mRNA, several microRNAs, namely miRNA-214 and miRNA-149, exhibit inhibitory properties, in contrast with long-noncoding RNAs, like lncRNA-H19 and CCAT2, which upregulates its expression. Following the translation, the FOXM1 protein may undergo several modifications, including phosphorylation, ubiquitination, SUMOylation, acetylation, and methylation [103].

FOXM1 is predominantly detected in progenitor cells and regenerating tissues. Nevertheless, it is also detected in malignancies promoting aberrant cell proliferation, migration, and genomic instability, the known hallmarks of cancer [104]. FOXM1 has been associated with cell migration, invasion, stemness, mesenchymal (MES) transition, and resistance to radiotherapy in gliomas. Zeng et al. demonstrated a positive correlation between *FOXM1* and *Abnormal Spindle-like Microcephaly (ASPM)* expression, a protein essential for normal mitotic spindle function in embryonic neuroblasts associated with poor outcome of glioma patients. ChIP assay and luciferase reporter analysis showed that FOXM1 wields *ASPM* expression via the direct binding to its promoter at −236 to −230 bp and −1354 to −1348 bp [105]. Furthermore, it has been demonstrated in TCGA glioma patient cohorts that *FOXM1* and *MYBL2* expression are linked in gliomas. The downregulation of *MYBL2* and *FOXM1* by siRNAs resulted in cell cycle arrest, apoptosis, and concomitantly abrogated the expression of certain EMT and invasion markers, such as N-cadherin and MMP-2. Taken together with inhibition studies aiming the Akt/FOXM1 signaling, these results propose that transcription factor MYBL2 functions as a key downstream component of the Akt/FOXM1 axis, promoting the progression of gliomas [106]. Moreover, FOXM1 is associated with resistance to radiation, since its inhibition with siomycin-A (SM) and concurrent radiotherapy mediated mitotic catastrophe in GBM cells. In addition, repression of the factor’s expression by SM and siRNAs revealed an attenuated expression of genes involved in DNA repair (*MRE11* and *RAD51*) and inhibited the Homologous Recombination (HR) pathway, an essential DNA double-stranded break (DSB) repair mechanism. In the same study, a physical interaction of FOXM1 with the phosphorylated state of STAT3 transcription factor was demonstrated under radiation, leading to the hypothesis that the two factors cooperate to establish radioresistance in GBM cells [107]. Senfter et al. showed that FOXM1 overexpression in tissue samples from medulloblastoma patients was a result of miRNA-4521 loss. This finding is of great importance, since restoration of this microRNA levels through transfection induced apoptosis via caspase 3/7 activation and regulated the proliferation and invasive abilities of several medulloblastoma cell lines [108]. Regarding MES transition, a hallmark of GBM, it was shown that FOXM1 binding to *A Disintegrin And Mettaloproteinase 17 (ADAM17)* promoter upregulates its expression and maintains the ADAM17/EGFR feedback loop that promotes mesenchymal transition in GBM [109]. Tao et al. demonstrated that SATB2 (AT-rich Binding Protein 2), which is a significant NMP (Nuclear Matrix-associated Protein), binds to the MAR sequence of *FOXM1* and recruits histone acetyltransferase CBP in order to activate its transcription. This mechanism that leads to the induction of *FOXM1* expression is present mostly in GSCs and is implicated in GBM progression [110].

The role of proteasome inhibitor (PI) Bortezomib in gliomas has been investigated by several research groups, although the mechanism behind its antitumor effects has not been fully understood [111,112,113]. A study investigating the chemotherapeutic role of Bortezomib and its underlying mechanism in gliomas, revealed a connection between the effect of the proteasome inhibitor and the Akt/FoxM1 signaling axis. By using cell viability, flow cytometry, and colony formation assays, they observed that low concentrations of Bortezomib abolished proliferation, colony formation, and spheroid growth and attenuated the stem cell phenotype of glioma cells through apoptotic mechanisms and cell cycle arrest. This agent also exhibited a synergy with TMZ and increased glioma cells susceptibility towards TMZ treatment both in vitro and in vivo. Furthermore, overexpression and knockdown experiments in glioma cells revealed that FoxM1 is a key target of Bortezomib, since its downregulation appeared to underlie the cytotoxic effects of the inhibitor. In addition, the antiapoptotic protein Survivin was linked to FoxM1 as a downstream effector. Overall, they proposed that Bortezomib exerts its chemotherapeutic effect through inhibition of the FoxM1–Survivin pathway, which is often found deregulated in HGG [114]. Furthermore, the physical compound Plumbagin (5-hydroxy-2-methyl-1,4-naphthoquinone), an active constituent of the roots of the medicinal plant *Plumbago zeylanica* L., was found to cause reduction of glioma tumor growth and cell proliferation, in vivo and in vitro via apoptotic pathways. Treatment with Plumbagin downregulated the expression of *FoxM1* and its downstream targets, *cyclin D1* and *Cdc25B*, while elevated the expression of *p21* and *p27*. These findings suggested that this natural compound may function against glioma progression through inactivation of FoxM1 [115].

### 2.6. ATF4 Transcription Factor

Activating transcription factor 4 (ATF4) belongs to a group of basic-region leucine zipper (bZIP) transcription regulators, which embody the CREB/ATF family. *ATF4* is located at the 22q13.1 locus of chromosome 22. The three open reading frames (uORFs) observed in human *ATF4* mRNA reside in the 5’ UTR foregoing its coding sequence are vital for the factor’s response under stressful and hypoxic conditions. At the protein level, ATF4 encompasses certain crucial motifs for its dimerization, stability, and binding to genes of interest. These motifs include an ODDD, the betaTrCP degradation recognition domain, an N-terminal TAD, and a DBD within the basic region at the C-terminal. Enclosed in the basic region, there is a sequence described as KKLKK that extends from amino acids 280 to 284 and plays an important role in nuclear targeting. Furthermore, ATF proteins mediate transcriptional regulation by recognizing the TGACGTCA consensus sequence at the promoter of their target genes [116,117].

*ATF4* is mostly known as a stress responsive gene whose expression gets upregulated during oxygen deprivation (hypoxia/anoxia), endoplasmic reticulum stress (UPR pathway), oxidative stress, and amino acid or nutrient destitution but still possesses a part in skeletal and eye development, autophagy, and hematopoiesis. ATF4 holds a dual role in cell homeostasis due to its ability to target either adaptive or stressful condition genes that promote long-term cell survival or proapoptotic genes. The outcome of ATF4 activation is context-dependent and associates with the protein partners that it interacts with or dimerizes. For instance, heterodimers of ATF4 with C/EBPβ or C/EBPγ provoke adaptation, whereas its dimerization with CHOP results in proapoptotic signaling by regulating *BCL2* and *BIM*. Noteworthy, ATF4 also forms heterodimers with members of AP-1 (FOS and JUN), C/EBP bZIP subfamilies, including its own family, and has the ability to homodimerize, although the homodimers do not represent a stable complex even when bound to DNA.

A dominant downstream event of many stress-induced signaling cascades is the phosphorylation of eukaryotic initiation factor 2 (eIF2a) on Ser51 of its α-subunit. A range of kinases involved in ER stress; amino acid limitation and UV exposure; viral infection; heme deprivation; and oxidative stress responses like PERK, GCN2, PKR, and HRI, respectively, are responsible for this modification. Although this alteration imposes a global suppression on protein synthesis, it heightens the translation of *ATF4* and some other mRNAs. Noteworthy, hypoxia has the same effect in *ATF4* translation, since, during such conditions, eIF2a is phosphorylated by an indirect mechanism that implicates UPR and PERK. However, ATF4 stability is also regulated via its ODDD independently of the peIF2a presence. The posttranslational control of ATF4 is coordinated by interactions with βTrCP and hypoxia inducible PHD3 proteins. Specifically, casein kinase-dependent phosphorylation of nuclear ATF4 on Ser219 at its βTrCP recognition motif leads to binding of the βTrCP protein and, ultimately, ubiquination and proteosomal degradation of the factor. This interaction can be enhanced by the accumulation of the negative charge in proximity with the recognition motif’s region caused by phosphorylation on Thr213, Ser224, Ser231, Ser235, and Ser248. On the contrary, the binding of PHD3 protein stabilizes the ATF4 structure, probably due to proline hydroxylation at the ODDD [116,117].

Tumor growth induces stress and nutrient deficiency, which mostly affect the cells at the center of the mass. Despite the risk of being driven to apoptosis, cancer cells frequently activate the upregulation of *ATF4* under such circumstances to survive the consequences of stressful conditions. *ATF4* expression was found elevated in the malignant types of gliomas and in the high-grade tumors correlated with poor overall patient survival [118,119]. ATF4 presence and accumulation has an impact on cell morphology, with engineered *ATF4* overexpressing glioma cells being bigger and displaying a polyplastic phenotype, while *ATF4* knockdown cells are smaller and display a spindle-like phenotype, with a maximal two membrane extensions compared to the controls [118,119]. In addition, colony formation assays demonstrated that *ATF4* expression promotes glioma cell proliferation and migration. The same study showed that the ATF4 factor is responsible for the regulation of *glutamate antiporter xCT (SLC7a11)* expression, which is a critical tumor-induced intoxication of the brain’s microenvironment, and glutamate secretion in human malignant glioma specimens. ATF4 overexpressing tumor cells release an excess of glutamate in the microenvironment, contributing to neurodegeneration and brain swelling. In addition, glutamate secretion and ATF4-mediated function of xCT represent a candidate mechanism for the promotion of angiogenesis in ATF4 overexpressing gliomas, besides the induction of *VEGF* and *HIF-1α* expression. The xCT pathway mediated by ATF4, lastly, confers glioma tumor resistance towards chemotherapy with TMZ [120].

As previously mentioned, ER stress can often be triggered by anticancer agents and lead to a UPR response, which bestows tumor cells greater tumorigenic abilities and drug-resistance. Dihydroartemisinin (DHA), an active byproduct of Artemisin (ART) that derives from the Chinese medicinal herb *Artemisia annua* L., exhibits anticancer properties through a not-so-typical form of cell death caused by the iron-dependent production of reactive oxygen species (ROS). Experimental evidence indicates that DHA results in glioma cell death. However, this mechanism activates PERK/ATF4 as a response to ER stress, which, in turn, activates the genes that make glioma cells resistant to DHA treatment. A promising approach to overcome this problem seems to be the concomitant treatment with DHA and PERK inhibitor I (GSK2606414) or siRNA-mediated silencing of *ATF4*, shown to enhance the cytotoxic effects of DHA [121]. Another natural product, Flavokawain B (FKB), has been shown to attenuate GBM cell growth via senescence and autophagy. FKB-induced autophagy was mediated by the ATF4-DDITR3 ER stress signaling pathway. Inhibitors of autophagy (3-MA or CQ) or knockdown of *ATF4* and other related genes were shown to switch the status of FKB-induced senescence to FKB-induced apoptosis in glioma cells [122].

## 3. Tumor Suppressor Transcription Factors

Several transcription factors have also been detected to be involved in tumor suppression by modulating gene expression. Among them, members of NFI, T-box, and NZF families have demonstrated a tumor-suppressive role in gliomas with targeting potential.

### 3.1. NFI Transcription Factors

The Nuclear Factor I (NFI or CTF) family encompasses proteins that participate both in viral DNA replication and in gene expression regulation as transcription factors. The four components of the family, NFIA, NFIB, NIFC, and NIFX, play a decisive role in CNS development, specifically in axon guidance and outgrowth and glial and neuronal cell differentiation, as well as neuronal migration. It is of foremost importance that the expression of NFIA and NFIB persists in mature astrocytes. NFI factors are components of several hormonal and signal transduction pathways orchestrated by insulin, TGF-β, cAMP, steroid hormones, vitamin B6, TNF-α, FSH, thyrotropin, etc. [123,124].

At the transcriptional level, up to nine unique variants per gene have been identified, produced by alternative splicing. The longest mRNA transcript of each member contains 11 to 12 exons. The splice variants are conserved at a 90% degree among species, while it is impressive that different variants have been established in the brain with unknown significance and functional role. Thereinafter, at the protein level, NFI factors contain a DNA-binding and dimerization domain at the N-terminal and a transcription modulation domain, which is implicated in the activation or repression of transcription at the C-terminal. Of importance, the parts of the protein’s structure that are conserved among the family members include the 200–220-amino acid-long DNA-binding domain and four cysteine residues within it. Three of the cysteine residues are important for the DNA-binding process, while the fourth accounts for the susceptibility of NFI factors to oxidative inactivation and redox control. The C-terminal domain is proline-rich and, as foretold, is responsible either for transactivation or repression of target genes transcription, depending on the promoter type, the cellular context, and interaction with coactivator proteins.

Although the mechanism determining whether a NFI factor is going to repress or activate transcription is not fully understood, one thing is certain, that all members need to form homo- or heterodimers in order to bind successfully to the common recognition sequence 5’-TTGGCXXXXXGCCAA-3’. The factors are also able to bind to the consensus half sites (TTGGC or GCCAA) at a lower affinity. The binding affinity is modulated by sequences close to the consensus and the configuration of the 5-nucleotide spacer region. Several posttranslational modifications of NFI proteins have been observed, including phosphorylation by cell division cycle 2 (CDC2) and JAK kinases, as well as N- or O-linked glycosylation [123,125].

The role of NFI factors in glial differentiation has led to investigation of their impact in the pathogenesis and progression of gliomas. In this area, the existing evidence is very contradicting, as NFI factors have been reported to promote glioma progression in some studies and suppress it in some others. Chen et al. demonstrated that NFIA and NFIB are co-expressed mainly in the same cells of GBM tumors and that their expression decreases as the tumor grade rises. This finding is expected, since these factors promote cell differentiation and high-grade tumors contain mostly undifferentiated cells. Furthermore, NFIA’s and NFIB’s presence were correlated with genes representing the mature astrocytic state. Co-staining with astrocytic (GFAP) and proliferation (Ki67) markers in GBM samples and cell lines showed that these TFs are associated to a nonproliferating and differentiated profile of cells expressing them. Overexpression of these factors was shown to be adequate for the switch of proliferative cells towards astrocytic differentiation in xenografts [124]. Another study linked the regulation of HEY1, which is a component of the Notch pathway participating in neural stem cell maintenance, with the expression levels of all four NFI family members. In particular, the regulator proteins bind to NFI-recognition sites located within 1 kb upstream of the HEY1 transcription site and negatively regulate its transcription. They further demonstrated via HEY1 knockdown that the effector was responsible for cell proliferation, increased cell migration, and neurosphere formation of GBM cells, being correlated with the expression of the brain neural stem/progenitor cell marker B-FABP [126]. Vo et al. identified a positive feedback loop between NFIB and calpain I that prevents GBM cell migration. The active state of NFI is considered the dephosphorylated form of the factor [127]. Dephosphorylation of NFIs is induced by Calcineurin phosphatase, which, in turn, is cleaved and activated by Calpain proteases. Another component, Calpastatin (CAST), regulates this axis serving as an endogenous inhibitor. It has been reported that the CAST gene is an NFI target in GBM and that differentially regulated NFI affects the levels of CAST variants at the transcriptional level. They observed that NFI-hyperphosphorylated GBM cells exhibited a decreased cytoplasmic CAST/Calpain 1 ratio, which caused elevated autolysis and activity of Calpain 1 in the cytoplasm. Within NFI-hypophosphorylated cells, the expression of NFIB drives differential subcellular cell localization of CAST and calpain, with the first being primarily in the cytoplasm and the latter in the nucleus. This resulted in increased Calpain 1 activity in the nucleus, which caused Calcineurin activation and, ultimately, the induction of NFIB dephosphorylation. Of great interest, the knockdown of either one or both of NFIB and Calpain 1 escalated the migration of GBM cells and upregulated the promigratory factors FABP7 and RHOA. Altogether, this positive feedback loop may abrogate GBM cell migration but has zero effect on cell survival [125]. Moreover, Chen et al. observed the tumor-suppressive role of NFIA/B after deletion of either NFI gene in established high-grade astrocytomas mice models, when tumor growth and aggression increased [128].

On the other hand, Yu et al. reported an enriched expression of NFIA in GBM that conferred to TMZ resistance and was associated with adverse patient outcome. Furthermore, they evaluated the functional role of NFIA in TMZ-resistant GBM. A concomitant increase in NFIA and NF-κΒ levels was observed in a TMZ-resistant cell line, in which suppression of NFIA resulted in NF-κΒ downregulation and re-sensitization in TMZ. They also demonstrated that NFIA expression was positively correlated to NF-κΒ promoter’s activity and that NFIA mediated the phosphorylation of NF-κΒ p65 unit on Ser536. IKKβ overexpression increased the levels of phosphorylated NF-κΒ, although this effect could be reversed, partially, by NFIA knockdown. This evidence indicates that NFIA promotes the resistance of GBM cells to TMZ through NF-κΒ phosphorylation [129].

Regarding NFI role in glioma therapy, NFIA has been identified to undergo regulation from microRNA miR-302b, which decreases glioma cell survival. Specifically, miR-302b suppresses NFIA expression, which, in turn, disrupts the dose-dependent binding of NFIA to IGFBP2 promoter and the subsequent enhancement of IGFBP2 downstream signaling. Hence, this particular miRNA seems to function through a regulatory loop that involves NFIA/IGFBP2 inhibition in order to induce death of glioma cells [130]. Additionally, miR-223 has been reported to downregulate NFIA expression and, ultimately, suppress glioma cell proliferation [131].

### 3.2. TBXT Transcription Factors

T-Box Transcription Factor T (TBXT), also known as Brachyury, is the founding member of the T-box protein family. The TBXT protein is located in the nucleus of notochord-derived cells, where it exerts its function as a transcription regulator of genes required in mesoderm formation and differentiation. With respect to development, Brachyury’s misexpression has been associated with several congenital defects, mainly neural tube defects, and the fact that homozygous embryos die after a few days of gestation.

T-box protein sizes range from 50 to 78 kDa and consist of two significant domains: the DNA-binding and a transcription modulator, whose position varies among the family members. The DNA-binding domain is often referred to as T-box, a relatively large region that occupies the one third of the entire protein. In general, the homology ratio of the T-box varies between proteins, but some specific residues within it remain 100% conserved. Despite the sequence variations of the DNA-binding domain, all T-box proteins bind to a specific DNA element, the palindromic T-site (TCACACCT). T-box proteins are able to activate or repress the transcription of their target genes and this regulation is guided by sequences at the C-terminal part [132,133].

Brachyury mostly functions as a transcriptional activator and has been found upregulated in several types of cancers, including breast, lung, colorectal, prostate, testicular, and gastrointestinal stromal tumors. In addition, supporting evidence has revealed its role in the promotion of tumor cell migration, invasion, and metastasis through EMT. Pinto et al. investigated the TBXT role in gliomas in two different studies. At first, they observed a differentiation of the mRNA levels in normal brain samples of both adults and children and glioma cell lines. TBXT expression was present in normal brains while absent or at low levels in gliomas and was inversely correlated with tumor grade, and TBXT loss was linked to the mesenchymal subtype of GBM. The latter is also associated with poor prognosis and indicates the tumor suppressive role of the protein in gliomas. They further investigated the reduced expression of TBXT in gliomas using RNA-sequencing, which revealed that, within the different anatomical structures of the tumor, TBXT is preferentially expressed in sections with a higher concentration of normal cells. Moreover, they demonstrated that Brachyury was able to increase the expression of several pro-apoptotic proteins and autophagy, which was confirmed by a decrease in cell viability in vitro and, consequently, in the tumor growth observed in vivo. In their second study, they demonstrated via gene-editing methods for the overexpression of the factor in glioma cells that TBXT-positive cells exhibit reduced invasive and migratory capability and stem cell features. Additionally, the same cells displayed a higher expression of differentiation markers. Furthermore, they used TMZ-resistant and TMZ-responsive cell lines and induced TBXT exogenous and endogenous activation through overexpression and retinoic acid treatment, respectively. This activation drove the TMZ sensitization of glioma-resistant cell lines. In conclusion, these novel findings highlighted the tumor suppressive nature of Brachyury in brain cancer, impairing gliomas’ aggressive features and progression [133,134].

### 3.3. MYT1 and MYTL1 Transcription Factors

The neural zinc-finger (NZF) protein family represents a small group of specific DNA-binding proteins, which includes the Myelin Transcription factor 1 (Myt1/NZF2), Myt1-like (Myt1l/NZF1), and Suppressor of Tumorigenicity 18 (ST18/NZF3) [135]. Overall, the members of this family are involved in CNS development, pancreatic function, and tumor progression. Myt1 was first identified due to its binding to proteolipid protein, also known as the promoter of the myelin gene, which is implicated in the structure and compaction of the myelin sheath that is located around the axons of the CNS [136]. Myt1 function is critical for the differentiation of endocrine islet cells in the pancreas [137]. It can also induce the proliferation and differentiation of oligodendrocytes, the myelin-forming cells of the CNS [138]. Myt1l function, combined with the activity of Ascl1 and Brnd2 transcription factors, has the ability to mold human stem cells directly into functional neurons [139,140]. At the same time Myt1l seems to attenuate the expression of non-neuronal genes ceasing non-neuronal cell fate [141]. Both Myt1 and Myt1l interact with the corepressor Sin3B and form complexes that recruit HDAC1 and HDAC2 to selected genes during CNS development [135].

The components of the NZF family contain two bundles (clusters) of C2H2 zinc fingers. The first cluster is spotted close to the middle of the protein and is composed of one pair of zinc fingers, while the other one consists of three pairs in Myt1l and four in Myt1 and ST18 at the C-terminal. In addition, Myt1 and Myt1l contain one more pair within the N-terminal. Hence, the Myt1 and Myt1l structure encompasses seven and six pairs of zinc fingers, respectively. The zinc fingers are notably conserved among the three NZF members but also exhibit high levels of similarity between them within each protein. The transcription factors recognize the consensus sequence AAAGTTT through interactions with their DNA-binding domain Cys-X4-Cys-X4-His-X7-His-X5-Cys (also termed the CCHHC domain) [136,142]. Of importance, the binding affinity is higher when both clusters interact with the consensus site. The DR9 element that includes two direct repeats of the consensus site nine pair bases apart from one another is considered as a preferred binding sequence [143]. Specifically, Myt1 has been shown to bind to DNA through its fifth zinc finger that fits into the major DNA groove and connects with the AGT site of the consensus motif [144].

Due to their participation in the evolvement of CNS and evidence of gene suppression, several studies have tried to decipher their role in gliomas. Myt1 has been shown to limit the growth of glioblastoma in a xenograft model by regulating the expression of RNA-binding protein Rbfox1 [145]. Melhuish et al. showed that the Myt1 or Myt1l factor restricts GBM cell proliferation upon reintroduction in vitro. They further examined the relative expression levels of MYT1 and MYT1L in human brain cancer datasets showing that MYT1L was expressed in a lower rate in oligodendroglioma, astrocytoma (grade III), and GBM compared to normal brains. In contrast, MYT1 levels were increased in oligodendroglioma and astrocytoma more than in a normal brain, but its expression in both astrocytoma and glioblastoma was significantly lower than in oligodendroglioma. Additionally, an analysis from the TCGA dataset of LGG revealed that both TFs are linked to the less aggressive subtype with IDH mutations and codeletion of the 1p and 19q chromosome regions. Besides the correlation with aggressiveness, Myt1 and Myt1l high expression levels indicate longer overall patient survival. The study identified a possible mechanism that confers to GBM progression, which involves the YAP1 transcriptional coactivator from the Hippo pathway, whose expression is normally repressed by Myt1 and Myt1l. YAP1 expression was revealed as a responsible factor for GBM cell proliferation. These facts are in accordance with Myt1 and Myt1l being downregulated in GBM while YAP1 was overexpressed [146].

At last, JLK1486, an 8-hydroxyquinoline–substituted benzylamine, was shown to induce anticancer activity in vivo through intravenous and oral administrative routes in a xenograft model and exhibited the same beneficial effects with those of TMZ. The benefits of JLK1486-treatment derive from its ability to activate various transcription factors, such as Myt1, STAT1, and peroxisome proliferator-activated receptor γ, in glioma cells. The activation of these TFs by JLK1486 had a cytostatic rather than a cytotoxic outcome of glioma cells [147].

## 4. Discussion

Overall, gliomagenesis and tumor progression rely on deregulation, among others, of the transcription factors emphasized in this review. These master regulators are responsible for glial differentiation, adaptation to stressful conditions, cell cycle control, and angiogenesis contributing to the aggressive nature and recurrence of the disease. They have also emerged as therapeutic targets and tools with prognostic values (Table 1). 

Current approaches for the investigation of their functional role are based mostly on glioma cell lines that are considered sometimes as an inadequate model for the understanding of the disease. Thus, patient-derived cells and orthotopic xenografts (PDX) may prove as more suitable models, since they resemble the original tumors and patient characteristics [150]. Since cancer is a multifunctional disease, the future goal is to unveil and describe the role of epigenetic modifications, noncoding RNAs, and transcription factors in the shaping of the cell’s regulatory network. Epigenetic modifications, for instance, regulate chromatin and the access of transcription factors to DNA. Gene expression and ChIP methods, next-generation sequencing, methylation profiling, and protein–protein interaction assays could help depict the activity and relationship between regulatory molecules and potentially provide for the stratification of patients based on molecular markers [70,151].

Future therapeutic approaches could be directed towards utilizing miRNAs against upregulated transcription effectors and antisense oligonucleotides as the means for the degradation of miRNAs that promote tumor development. Furthermore, it is important in this matter to increase the stability and optimize their delivery systems in order to decrease nonspecific target effects [152]. Moreover, the rapid development of nanoparticles technology could prove useful in the treatment of gliomas. For instance, nanoparticles could be used to deliver inhibitors against oncogenic TFs or molecules inducing the expression of tumor suppressive TFs [153]. In addition, gene therapy in the form of either gene addition or gene editing should be included in the treatment in order to change the way that a deregulated protein is produced, something that could benefit patients from a personalized angle [154]. These prospective treatments could be used alone or in combination with the standard TMZ therapy or other therapeutic agents to enhance the antitumor effects. In conclusion, there is an emerging need for diagnostic/prognostic biomarkers, molecular profiling, and targeted therapy that would help surpass the heterogeneity mediating gliomas progression and treatment resistance.

## Figures and Tables

**Table 1:** Candidate compounds and molecules that interact either directly or indirectly with glioma-related TFs. Structures and relevant information of several TF-related candidate compounds that could potentially be deployed in the battle against gliomas.

**Structure**	**Information**		**Structure**	**Information**	
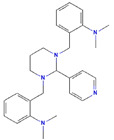	Molecular Formula:	C_27_H_35_N_5_	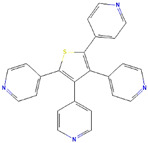	Molecular Formula:	C_24_H_16_N_4_S
	Compound Name:	GANT-61		Compound Name:	GANT-58
	PubChem CID:	421610		PubChem CID:	253078
	Target:	GLI		Target:	GLI
**Structure**	**Information**		**Structure**	**Information**	
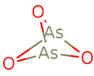	Molecular Formula:	As_2_O_3_	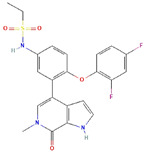	Molecular Formula:	C_22_H_19_F_2_N_3_O_4_S
	Compound Name:	Arsenic Trioxide		Compound Name:	ABBV-075/ Mivebresib
	PubChem CID:	14888		PubChem CID:	71600087
	Target:	GLI		Target:	GLI
**Structure**	**Information**		**Structure**	**Information**	
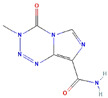	Molecular Formula:	C_6_H_6_N_6_O_2_	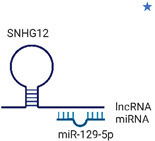	Molecular Formula:	-
	Compound Name:	TMZ/ Temozolomide		Compound Name:	SNHG12 and miR-129-5p
	PubChem CID:	5394		PubChem CID:	-
	Target:	E2F		Target:	E2F
**Structure**	**Information**		**Structure**	**Information**	
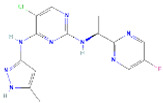	Molecular Formula:	C_14_H_14_ClFN_8_	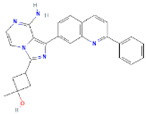	Molecular Formula:	C_26_H_23_N_5_O
	Compound Name:	AZD1480		Compound Name:	Linsitinib
	PubChem CID:	16659841		PubChem CID:	11640390
	Target:	STAT3		Target:	STAT3
**Structure**	**Information**		**Structure**	**Information**	
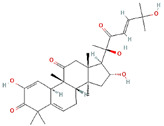	Molecular Formula:	C_30_H_42_O_7_	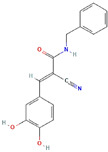	Molecular Formula:	C_17_H_14_N_2_O_3_
	Compound Name:	JSI-124/ Cucurbitacin I		Compound Name:	AG490/ Tyrphostin B42
	PubChem CID:	5281321		PubChem CID:	5328779
	Target:	STAT3		Target:	STAT3
**Structure**	**Information**		**Structure**	**Information**	
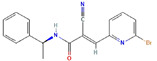	Molecular Formula:	C_17_H_14_BrN_3_O	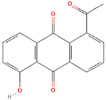	Molecular Formula:	C_16_H_10_O_4_
	Compound Name:	WP1066		Compound Name:	LLL3
	PubChem CID:	11210478		PubChem CID:	16051915
	Target:	STAT3		Target:	STAT3
**Structure**	**Information**		**Structure**	**Information**	
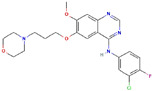	Molecular Formula:	C_22_H_24_ClFN_4_O_3_	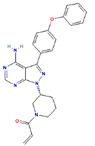	Molecular Formula:	C_25_H_24_N_6_O_2_
	Compound Name:	Gefitinib		Compound Name:	Ibrutinib
	PubChem CID:	123631		PubChem CID:	24821094
	Target:	STAT3		Target:	STAT3
**Structure**	**Information**		**Structure**	**Information**	
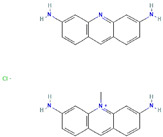	Molecular Formula:	C_27_H_25_ClN_6_	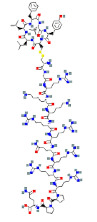	Molecular Formula:	C_111_H_188_N_42_O_24_S_2_
	Compound Name:	Acriflavine		Compound Name:	TAT-cyclo-CLLFVY
	PubChem CID:	443101		PubChem CID:	72192490
	Target:	HIF		Target:	HIF
**Structure**	**Information**		**Structure**	**Information**	
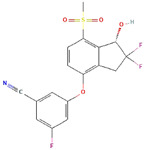	Molecular Formula:	C_17_H_12_F_3_NO_4_S	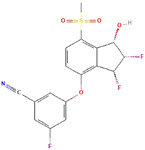	Molecular Formula:	C_17_H_12_F_3_NO_4_S
	Compound Name:	PT2385		Compound Name:	PT2977/ Belzutifan
	PubChem CID:	91754484		PubChem CID:	117947097
	Target:	HIF		Target:	HIF
**Structure**	**Information**		**Structure**	**Information**	
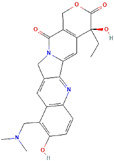	Molecular Formula:	C_23_H_23_N_3_O_5_	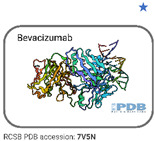	Molecular Formula:	C_6638_H_10160_N_1720_O_2108_S_44_
	Compound Name:	Topotecan		Compound Name:	Bevacizumab/ Anti-VEGF Monoclonal Antibody
	PubChem CID:	60700		PubChem CID:	178103377
	Target:	HIF		Target:	HIF
**Structure**	**Information**		**Structure**	**Information**	
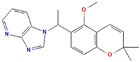	Molecular Formula:	C_20_H_21_N_3_O_2_	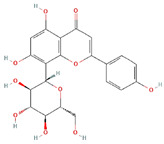	Molecular Formula:	C_21_H_20_O_10_
	Compound Name:	103D5R		Compound Name:	Vitexin
	PubChem CID:	11267663		PubChem CID:	5280441
	Target:	HIF		Target:	HIF
**Structure**	**Information**		**Structure**	**Information**	
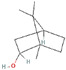	Molecular Formula:	C_10_H_18_O	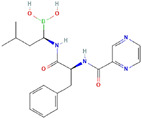	Molecular Formula:	C_19_H_25_BN_4_O_4_
	Compound Name:	Borneol		Compound Name:	Bortezomib
	PubChem CID:	64685		PubChem CID:	387447
	Target:	HIF		Target:	FOXM1
**Structure**	**Information**		**Structure**	**Information**	
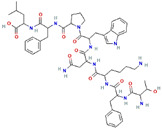	Molecular Formula:	C_54_H_73_N_11_O_11_	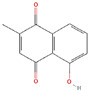	Molecular Formula:	C_11_H_8_O_3_
	Compound Name:	Survivin		Compound Name:	Plumbagin
	PubChem CID:	71464394		PubChem CID:	10205
	Target:	FOXM1		Target:	FOXM1
**Structure**	**Information**		**Structure**	**Information**	
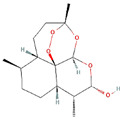	Molecular Formula:	C_15_H_24_O_5_	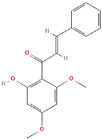	Molecular Formula:	C_17_H_16_O_4_
	Compound Name:	Dihydroartemisinin		Compound Name:	Flavokawain B
	PubChem CID:	3000518		PubChem CID:	5356121
	Target:	ATF4		Target:	ATF4
**Structure**	**Information**		**Structure**	**Information**	
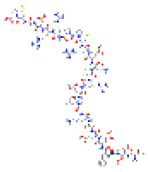	Molecular Formula:	C_124_H_205_N_39_O_39_S_2_	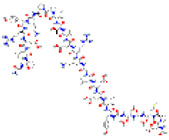	Molecular Formula:	C_142_H_230_N_36_O_44_S
	Compound Name:	Calcineurin Autoinhibitory Peptide/ PP2B-AIP		Compound Name:	Calpastatin/ Calpastatin Peptide B27-WT
	PubChem CID:	16219117		PubChem CID:	90488788
	Target:	NFI		Target:	NFI
**Structure**	**Information**		**Structure**	**Information**	
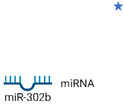	Molecular Formula:	-	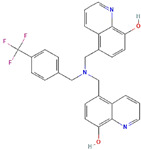	Molecular Formula:	C_28_H_22_F_3_N_3_O_2_
	Compound Name:	miR-302b		Compound Name:	JLK1486
	PubChem CID:	-		PubChem CID:	25268906
	Target:	NFI		Target:	MYT1 and MYTL1

The structures shown in this table and their relevant information were extracted from PubChem (https://pubchem.ncbi.nlm.nih.gov, accessed on 22 February 2022) [148]. Protein tertiary structure derives from the RCSB Protein Data Bank (PDB; http://www.rcsb.org/pdb/, accessed on 22 February 2022) [149]. Figures with the blue star symbol on the top right corner were created by BioRender.com, accessed on 23 February 2022.

## Data Availability

Not applicable.

## Abbreviations

ACF: Acriflavine; ADAM17: A Disintegrin And Metalloproteinase 17; ART: Artemisin; ASPM: Abnormal Spindle-like Microcephaly; As2O3: Arsenic Trioxide; ATF4: Activating transcription factor 4; ATRX: alpha-thalassemia/mental retardation X-linked; BCAAs: branched-chain amino acids; bHLH: basic helix-hoop-helix; BMX: bone marrow and X-linked; BRD4: bromodomain-containing protein 4; cAMP: cyclic adenosite monophosphate; CAST: Calpastatin; CC: coiled-coil; CDC2: cell division cycle 2; CK1: casein kinase 1; CNS: central nervous system; CTAD: C-terminal transactivation domain; DAXX: Death domain Associated protein; DBD: DNA binding domain; DHA: Dihydroartemisinin; DIM: dimerization; DSB: DNA-double strand break; eIF2a: eukaryotic initiation factor 2; EMT: epithelial to mesenchymal transition; ERE: Estrogen Responsive Element; FKB: Flavokawain B; FOXM1: Forkhead Box M1; GBM: Glioblastoma; GLI: Glioma-Associated Oncogene; GliA: Gli activator; GliFL: full-length glioma-associated oncogene; GliR: Gli repressor; GSCs: glioma stem cells; GSK3: glycogen synthase kinase-3; HBO: Hyperbaric oxygen; HGG: high-grade gliomas; Hh: Hedgehog; HIFs: Hypoxia-inducible transcription factors; HR: Homologous Recombination; HRE: HIF-responsive elements; IDH: Isocitrate Dehydrogenase; iNOS: inducible nitric oxide synthase; IR: ionizing radiation treatment; LGGs: low-grade gliomas; LZ: leucine zipper; MB: Medulloblastoma; MB: marked box; MES: mesenchymal; MMP: matrix metalloproteinase; Myt1: Myelin Transcription factor 1; Myt1l: Myt1-like; NFI: Nuclear Factor I; NF1: Neurofibromatosis type 1; NHAs: normal human astrocytes; NMP: Nuclear Matrix-associated Protein; NPs: nanoparticles; NRD: N-terminal repressor domain; NTAD: N-terminal translocation domain; NZF: neural zinc-finger; ODDD: oxygen dependent degradation domain; PAS: per-ARNT-Sim; PC: primary cilia; PI: proteasome inhibitor; PKA: protein kinase; PKCε: Protein Kinase C epsilon; PHD: prolyl hydroxylase domain protein; PTEN: Phosphatase and Tensin homolog; PTCH: Patched Receptors; p53: tumor protein p53; RB: Retinoblastoma; ROS: reactive oxygen species; SATB2: AT-rich Binding Protein 2; SH2: Src Homology 2; siRNA: small-interfering RNA; SM: siomycin-A; Smo: Smoothened Receptor; STAT3: Signal Transducer and Activator of Transcription 3; ST18: Suppressor of Tumorigenicity 18; Sufu: Suppressor of fused homolog; TAD: transactivation domain; TBXT: T-Box Transcription Factor T; TMZ: temozolomide; TERT: Telomerase Reverse Transcriptase; TFs: transcription factors; TFDP1: transcription factor dimerization partner family; uORFs: upstream open reading frames; VHL: Von Hippel-Lindau; 2HG: 2-hydroxyglutarate.
